# Moderne Bildgebung des Lungenhilus: Anatomie, Pathologien, Pitfalls

**DOI:** 10.1007/s00117-022-01103-6

**Published:** 2023-01-10

**Authors:** Daniel Kronenberg, Thomas Frauenfelder

**Affiliations:** Institut für Diagnostische und Interventionelle Radiologie, Universitätsspital Zürich, Rämistr. 100, 8091 Zürich, Schweiz

**Keywords:** Röntgenbildgebung, Thorax, Computertomographie, Lungenhilus, Muster, Radiography of the lung, Thorax, Computed tomography, Hilum of the lung, Patterns

## Abstract

**Hintergrund:**

Die moderne medizinische Bildgebung ist ein wichtiger Teil der effizienten ambulanten und stationären Patientenversorgung. Die konventionelle Röntgenaufnahme und die Computertomographie (CT) gehören zu den am häufigsten durchgeführten radiologischen Untersuchungen. Die Radiologie ermöglicht eine zielorientierte Präzisionsmedizin.

**Fragestellung:**

Ziel dieses Beitrags ist eine Übersicht über die Anatomie und die häufigsten Pathologien am Lungenhilus (LH), einer wichtigen Schnittstelle elementarer Strukturen des Thorax. Außerdem werden wichtige Zeichen und Muster zur Bildinterpretation in verschiedenen Modalitäten betrachtet.

**Ergebnisse:**

Eine genaue Kenntnis der Anatomie, der bildgebenden Zeichen pathologischer Veränderungen und *Pitfalls* in der konventionellen Röntgenaufnahme und in der sensitiveren Schnittbildgebung ist essenziell, um eine zielgerichtete Patientenversorgung zu unterstützen.

**Schlussfolgerung:**

Das konventionelle Röntgenbild ist günstig und schnell verfügbar. Es eignet sich sehr gut zum Pathologie-Screening, u. a. am Lungenhilus. Die Schnittbildgebung präzisiert aufgrund der deutlich besseren Differenzierbarkeit anatomischer Strukturen die Diagnostik.

## Abstract (en)

**Background:**

Modern medical imaging is a key component of efficient in- and out-patient precision medicine. Conventional radiography and computer tomography scans (CT) are among the most frequent radiologic exams. Medical imaging plays a key role in target-oriented medicine.

**Objectives:**

The purpose of this article is to review the anatomy of the hilum of the lung and its most frequent associated pathologies since it is an important gateway of elementary structures of the thorax. Important signs and patterns for image interpretation in different modalities are also reviewed.

**Results:**

Thorough knowledge of anatomy, signs, and patterns of pathologies especially in conventional radiography and pitfalls of the more sensitive cross-sectional imaging is essential to support target-oriented patient care.

**Conclusion:**

Conventional radiography is affordable and readily available. It is very suitable for pathology screening, i.e., at the hilum of the lung. Cross-sectional imaging specifies diagnostics due to superior anatomic discrimination.

Am Lungenhilus (LH), der Lungenverankerung im Mediastinum, finden sich die zentralen Strukturen der Lungen. Die moderne medizinische Bildgebung mittels konventioneller Röntgenuntersuchung und Schnittbildverfahren ermöglicht es, Veränderungen des LH sichtbar zu machen. So können Rückschlüsse auf verschiedenste pulmonale und kardiovaskuläre Erkrankungen, wie die Sarkoidose, einen zentralen Lungentumor und eine pulmonale Hypertonie, gezogen werden. Dieses nichtinvasive Verfahren unterstützt eine schnelle und gezielte, interdisziplinäre Diagnostik, die vor allem in der Notaufnahme und der stationären Patientenversorgung heutzutage nicht mehr wegzudenken ist. Die wichtigsten bildgebenden Zeichen und auch einige *Pitfalls* sollen im Folgenden erörtert werden.

## Anatomie

Das Wort *Hilus* kommt aus dem Lateinischen und bedeutet „ein kleines Ding“. Obwohl sich dieser Begriff ursprünglich auf die Vertiefung eines Samens oder Samenkorns bezogen haben könnte, wird er auch zur Beschreibung der Vertiefung oder Grube an dem Teil eines Organs, in den die Gefäße und Nerven eintreten, verwendet [4]. Die Fleischner Society definiert in ihrem Glossary den Hilus der Lunge wie folgt: „It is the site on the medial aspect of the lung where the vessels and bronchi enter and leave the lung“ [9]. Die radiologische Definition des pulmonalen Hilus ist die von den Bronchien und Blutgefäßen selbst verursachte Verschattung. Die normale hiläre Opazität besteht überwiegend aus Pulmonalarterien (PA) und Pulmonalvenen (PV) [4].

### Atemwege und Gefäße

**Figure 1:**
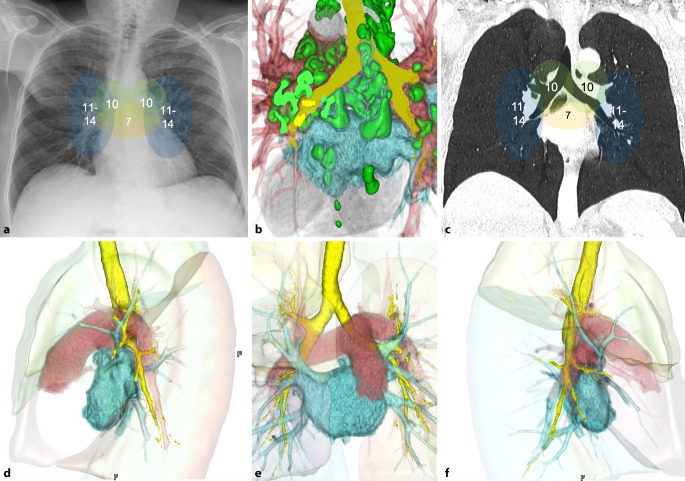

Entsprechend der unterschiedlich konfigurierten Lungenflügel ergeben sich Unterschiede im Aufbau des linken und rechten LH. Umgeben durch Pleura finden sich in beiden Hili vaskuläre Strukturen, Bronchial- und Lymphwege sowie Nerven- und Areolargewebe. Die aus dem Truncus pulmonalis und letztlich dem rechten Ventrikel entspringende rechte PA ist häufig etwas kaliberstärker als die Gegenseite und verläuft dorsal der Aorta thoracalis ascendens, der V. cava superior und der rechten oberen Lungenvene zum rechten LH. Linksseitig ist die PA kürzer und weiter nach dorsokranial gerichtet (Abb. 1). Entspringend aus dem Truncus pulmonalis, verläuft sie unter dem Aortenbogen hindurch, ventral der Aorta thoracalis descendens und kranial des Hauptbronchus nach links zum LH. Am Austritt aus dem Perikard ist sie über das Ligamentum arteriosum mit der Aorta verbunden. Beide PA teilen sich im LH entsprechend der pulmonalen Lobär- und Segmentanatomie auf und verlaufen mit den Bronchien als Endarterien in das Kapillarbett. Im rechten Hilus verlaufen zwei PA vor zwei Lobärbronchien. Aus dem alveolären Kapillarbett fließt das mit Sauerstoff angereicherte Blut über jeweils einen kranialen und kaudalen Venenstamm, den PV, von dorsal in den linken Vorhof. Der N. phrenicus verläuft in beiden LH ventral der Lungenwurzel.

### Lymphknoten

Über den LH drainiert auch das lymphatische System aus der Lunge. Die hilären Lymphknoten spielen hierbei eine wichtige Rolle als Wächterlymphknoten, sei es für entzündliche, infektiöse oder neoplastische Erkrankungen. Da die akkurate Erhebung der Lymphknotenbeteiligung ein wichtiger Teil in der Therapie des Lungenkarzinoms ist, hat die International Association for the Study of Lung Cancer (IASLC) eine Klassifikation zur standardisierten Bezeichnung der mediastinalen Lymphknotenstationen herausgegeben (Abb. 1). Nach der IASLC-Klassifikation sind die hilären Lymphknoten entlang der Hauptbronchien die Station 10R und 10L, R für rechts und L respektive für links [21]. Die hilären Lymphknoten findet man zahlenmäßig am häufigsten an der bronchialen Bifurkation, zwischen den Hauptbronchien und den Pulmonalgefäßen.

Das Röntgenbild ist zur Beurteilung nicht sehr sensitiv. Konventionell radiologisch zeigen sich vergrößerte Lymphknoten erst ab einer deutlichen Vergrößerung u. a. durch ein verbreitertes Mediastinum und verbreiterte LH. Zum Teil lassen sich die Lymphknoten auch an Verkalkungen erkennen. In der CT können Lymphknoten genauer dargestellt werden. Um diese jedoch von den Pulmonalgefäßen abgrenzen zu können, ist die Verwendung von Kontrastmittel (KM) essenziell [25]. In der Computertomographie (CT) und Magnetresonanztomographie (MRT) werden Lymphknoten anhand ihrer Form und Größe beurteilt. In der Hybridbildgebung wird zusätzlich die metabolische Aktivität bewertet. Ab einem Kurzachsendurchmesser von > 10 mm spricht man von pathologisch vergrößerten Lymphknoten. Das Fehlen eines Fetthilus sowie eine rundliche Form gelten als suspekt.

#### Merke.

Der Lungenhilus ist eine wichtige Schaltstelle im Thorax.

## Pathologien

### Systemische Erkrankungen des peribronchovaskulären Interstitiums

Erkrankungen, die das lymphatische System betreffen, können zu Veränderungen entlang des peribronchovaskulären Interstitiums, dem lymphatischen Abflussweg der Lungen, und zu Lymphknotenvergrößerungen führen. Häufig zeigt sich eine Weichteilvermehrung um die Bronchien und die Pulmonalgefäße. Die Genese kann sowohl maligne als auch benigne sein. Typische Beispiele sind die thorakale Sarkoidose, eine metastasierte Krebserkrankung, die Pneumonie und die Pneumokoniose [17]. Einige damit einhergehende Erkrankungen werden im Folgenden besprochen.

#### Lymphom

Das Lymphom ist eine von Lymphozyten ausgehende Neoplasie. Sie kann sich nodal und auch extranodal manifestierten. Im Röntgen-Thorax kann sich eine scharf begrenzte Verbreiterung der LH zeigen; zum Teil kann diese lobuliert erscheinen. Konventionell radiologisch ist eine Diagnosestellung jedoch nicht möglich. In der Schnittbildgebung kann die primär unspezifische Verbreiterung der Hili differenziert werden, und vergrößerte, teils eingeschmolzene Lymphknoten können von anderen hilären Strukturen abgegrenzt werden. Lymphknotenverkalkungen sind nicht typisch und treten eher nach Radiotherapie auf [3]. Multiple, deutlich vergrößerte Lymphknoten an beiden LH schließen die Differenzialdiagnose Lymphom bereits ein, jedoch fließt die Beurteilung weiterer Lymphknotenstationen supra- und infradiaphragmal, des Lungenparenchyms, der Leber und Milz bei der Erstellung einer Verdachtsdiagnose mit ein.

#### Sarkoidose

**Figure 2:**
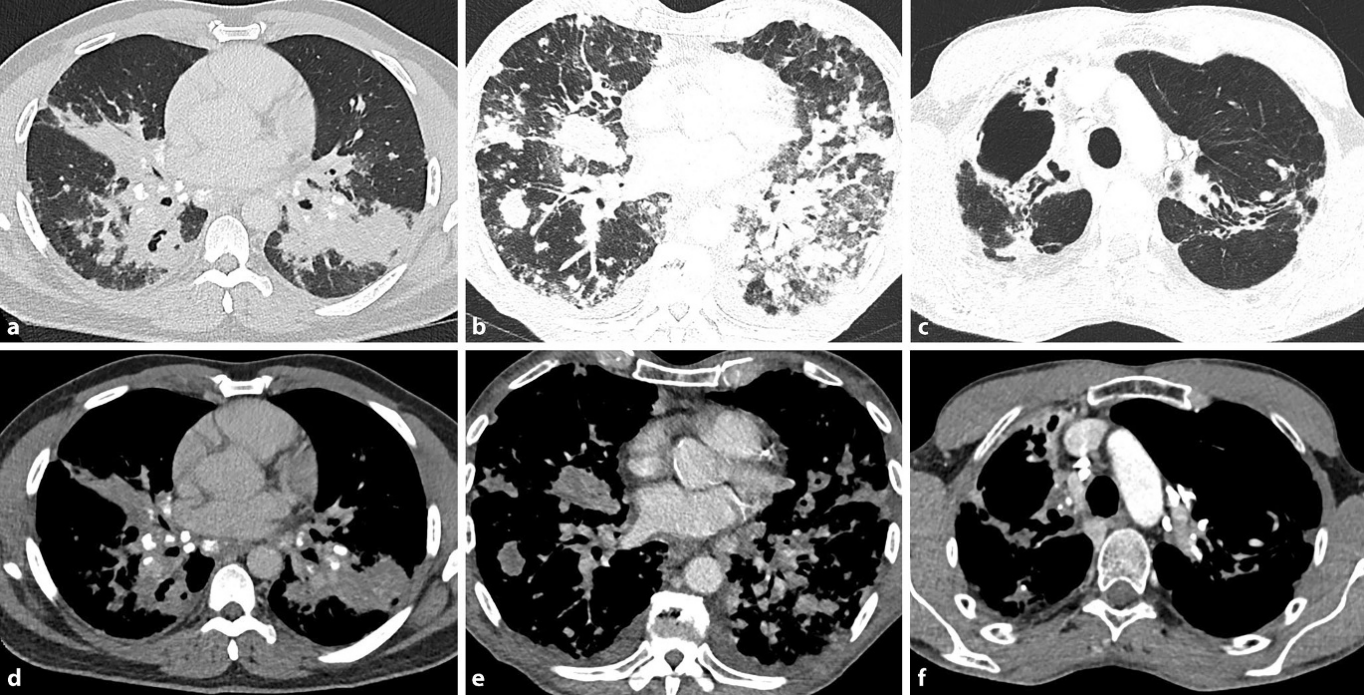

Die Sarkoidose ist eine multisystemische, nichtverkäsende granulomatöse Erkrankung unklarer Ätiologie. Die pulmonale und mediastinale Sarkoidose ist ein häufiger Manifestationsort und in der Regel von einer bihilären Lymphadenopathie zusätzlich zu den pulmonalen Veränderungen gekennzeichnet, welche sich entlang des lymphatischen Abflussweges ausbreiten (Abb. 2; [17]). Bei chronischer Genese können die Lymphknoten stippchenförmig, amorph oder eierschalenartig verkalken [16]. Diese Verkalkungen sind jedoch unspezifisch und man findet sie sowohl bei der chronischen Sarkoidose als auch beim radiotherapierten Lymphom [3]. Konventionell radiologisch zeigt sich die Lymphadenopathie in verbreiterten Hili ohne eine Alteration der Herzsilhouette. Durch das Garland- oder auch 1‑2-3-Zeichen lässt sich die pulmonale Sarkoidose von einem Lymphom unterscheiden [5]. Es beschreibt eine rechts-hiläre, links-hiläre und rechtsparatracheale Verbreiterung. Die Schnittbildung ist vor allem zur Darstellung der pulmonalen Veränderungen durch die anatomische Auflösung der konventionellen Radiologie überlegen. Die CT ist aber auch bei der Diagnose der typischen, teils verkalkenden, bihilären Lymphadenopathie deutlich sensitiver. Insbesondere die Sarkoidose, aber auch Lymphome, können zu einer hämodynamisch relevanten Komprimierung der PA führen und zu einem Druckanstieg im rechten Ventrikel.

### Erkrankungen der pulmonalen Gefäße

**Figure 3:**
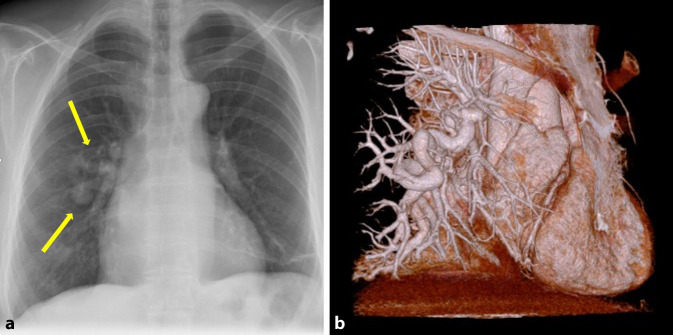

Zu den wichtigsten pathologischen Veränderungen der Pulmonalgefäße zählen insbesondere die akute Lungenembolie sowie die pulmonalvenöse (PVH) und pulmonalarterielle Hypertonie (PAH; [17]). Raritäten sind z. B. auch arteriovenöse Malformationen (Abb. 3). Die Schnittbildgebung ist der konventionellen Röntgenaufnahme in Sensitivität und Spezifität überlegen. PA und PV lassen sich genau unterscheiden, eine Gefäßerweiterung exakt quantifizieren und Verkalkungen bei chronischen Formen einer PAH einfacher erkennen.

#### Pulmonale Hypertonie

##### Pulmonalarterielle/präkapilläre Hypertonie.

Die PAH ist als Druckanstieg > 20 mmHG in den PA definiert [12, 20]. Die Ätiologie der PAH ist häufig idiopathisch. Die PAH wird gemäß der Nizza-Klassifikation in 5 Klassen eingeteilt [24]: (1) Idiopathische PAH, (2) PAH aufgrund von Linksherzerkrankungen, (3) PAH wegen Lungenerkrankungen, (4) chronisch-thromboembolische PAH (CTEPH) und (5) PAH aufgrund unklarer systemischer und multifaktorieller Ursachen.

Konventionell radiologische Zeichen eines erhöhten präkapillären/PA-Drucks können ein vergrößerter rechter Vorhof, vergrößerte, jedoch scharf abgrenzbare PA (Fleischer-Zeichen) und davon abgehende, sehr feine perihiläre Gefäßzeichnung (Knuckle-Zeichen) sein. CT-morphologisch gibt es weitere Zeichen, die auf eine PAH hindeuten: eine Erweiterung des Truncus pulmonalis > 29 mm [8, 19], erweiterte PA, das Carina-cross-over-Zeichen (Kreuzung von rechter PA und Carina) und pathologische Veränderungen des rechten Ventrikels im Sinne einer Dilatation und Hypertrophie (Myokarddicke > 5 mm).

**Figure 4:**
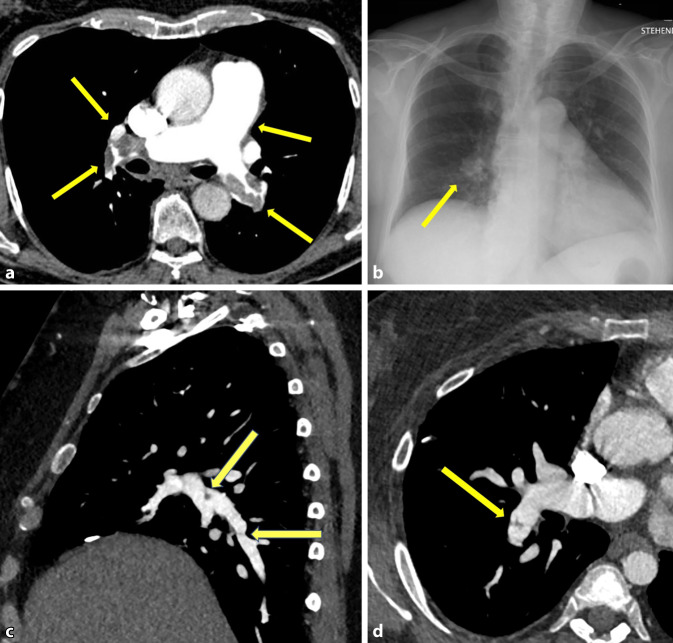

Eine aus radiologischer Sicht wichtige Form der PAH ist die CTEPH. Bei dieser Form finden sich im konventionellen Röntgenbild oft sekundäre Zeichen wie periphere keilförmige Konsolidationen oder streifige Dystelektasen. Schnittbildgebend lassen sich wandständige Verkalkungen der PA, als Hinweis auf eine chronische Genese, besser als im Röntgen-Thorax abgrenzen. Kaliberunregelmäßigkeiten der PA mit intravasalen Netzbildungen aufgrund von nicht vollständig abgebauten/rezidivierenden Emboli und eine pulmonale Mosaikperfusion sind typische Zeichen, die sich in der CT finden (Abb. 4; [26]). Gerade bei der Beurteilung der CTEPH spielt die MRT zunehmend eine gewichtige Bedeutung: dies zwar nicht in der Darstellung von Fibrinnetzen oder thrombotischen Auflagerungen, jedoch als „one-stop shop“ zur simultanen Beurteilung der Perfusion des Parenchyms, der kardialen Funktion und im beschränkten Maße auch der Morphologie [13].

##### Merke.

Sowohl schnittbildgebend als auch konventionell radiologisch lässt sich das Lungenödem nicht einfach von einer atypischen Pneumonie unterscheiden.

##### Pulmonalvenöse/postkapilläre Hypertonie.

Das Lungenödem, als Maximalausprägung der PHV, ist ein wichtiges Zeichen der akuten Herzinsuffizienz, welches sich bildgebend darstellen lässt [15]. Der erhöhte postkapilläre/pulmonalvenöse Druck kann sich im Röntgen-Thorax wie folgt manifestieren: (1) erweiterter vaskulärer Pedikel (Mediastinum oberhalb des Aortenbogens), (2) basoapikale Umverteilung, (3) periphere PV apikal ≥ periphere PV basal, (4) vergrößerter, im Gegensatz zur PAH deutlich unscharf abgrenzbarer LH, (5) bronchiales Cuffing und (6) periphere Curley-B-Linien bis hin zum (7) Vollbild des Lungenödems mit Pleuraergüssen (PE). Es gibt einige Unterschiede in der Liegendaufnahme aufgrund des hydrostatischen Drucks: Es gibt keine basoapikale Umverteilung und PE laufen nicht von den Randwinkeln nach kranial aus. Im Liegen verteilen sich PE dorsal. Diese flächige Transparenzminderung über der gesamten Lunge ist leicht zu übersehen bzw. falsch zu interpretieren.

Computertomographisch konnten fünf grundlegende Zeichen des Lungenödems identifiziert werden: (1) ein vergrößertes Herz, (2) bilateral verdickte Interlobärsepten, (3) bilaterale PE, (4) bilateral verbreiterte periphere Gefäße und (5) bilaterale „ground-glass-artige“ Opazitäten. Je mehr dieser Zeichen vorliegen, desto größer ist die Wahrscheinlichkeit eines Lungenödems [15].

**Figure 5:**
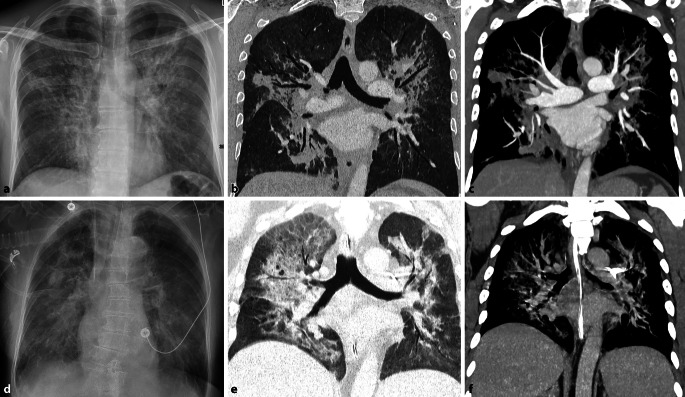

Sowohl schnittbildgebend als auch konventionell radiologisch ist die PHV bzw. das Lungenödem nicht einfach von einer atypischen Pneumonie zu unterscheiden (Abb. 5). Jedoch breiten sich die Zeichen der PVH im Gegensatz zur Pneumonie von zentral nach peripher aus. Weitere in der Computertomographie (CT) ersichtliche Zeichen wie Ansarka und kardiale Pathologien deuten eher in Richtung einer kardialen Genese.

##### Merke.

Mittels der weit verbreiteten CT lässt sich eine akute Lungenembolie (LE) schnell und mit hoher Sensitivität diagnostizieren. Multiplanare Rekonstruktionen (MPR) sind zum Erkennen von Netzen und Bändern bei der leicht übersehbaren chronischen LE hilfreich.

#### Lungenembolie

Bei der Lungenembolie verschließen Emboli meist mehrere PA. Durch verschlossene PA ist der Gasaustausch in der Lunge eingeschränkt, und es kann zu einer relevanten Hypoxämie kommen. Außerdem kann sie zu einer Rechtsherzbelastung führen, da das rechte Herz akut gegen einen erhöhten Widerstand anpumpen muss. Die CT ist die Methode der Wahl, um eine LE zu diagnostizieren. Durch die intravenöse (i.v.) Gabe von jodhaltigem Kontrastmittel (KM) können die PA dargestellt werden. Eine LE kann die PA ganz oder partiell verschließen. Das Polo-Mint-Zeichen beschreibt eine umspülte Kontrastmittelaussparung in der axialen Ebene der kontrastmittelgestützten CT, einen partiellen Verschluss [3].

Die Radiologin bzw. der Radiologe evaluiert das Ausmaß der LE: Wie viele Lungenlappen sind betroffen, wie ist die Ausdehnung von peripher nach zentral, und gibt es Zeichen der Rechtsherzbelastung? Ein erweiterter Truncus pulmonalis (Durchmesser > 27 mm bei Frauen, > 29 mm bei Männern), ein erweiterter rechter Ventrikel (RV/LV-Ratio > 1) zusammen mit einem „D-shape“ des linken Ventrikels sowie ein Rückstau des KM in thorakale und Lebervenen können hinweisend auf eine Rechtsherzbelastung sein [10, 14, 22, 28].

Konventionell radiologisch gibt es verschiedene, heutzutage kaum genutzte Zeichen, die auf eine LE hindeuten können. Beschrieben sind das Fleischner-Zeichen, das Palla-Zeichen, das Chang-Zeichen, das Knuckle-Zeichen, der Hampton-Hump, das Westermark-Zeichen und der PE als Begleitzeichen. Jedoch hat das konventionelle Röntgenbild aufgrund der geringen Sensitivität keine relevante Rolle in der Diagnostik der LE. Nichtsdestotrotz wird die konventionelle Röntgenuntersuchung bei den „ACR-Appropriateness Criteria“ unverändert mit „9 = usually appropriate“ gewertet, da sie dem Ausschluss anderer Pathologien dient [11].

Die MRT hat in den letzten Jahren deutlich an zeitlicher und örtlicher Auflösung gewonnen. Sie spielt jedoch bei der Lungenemboliediagnostik nach wie vor eine Nebenrolle und wird vor allem bei Schwangeren eingesetzt [1].

### Erkrankungen der Atemwege

Verschiedene benigne oder maligne Erkrankungen können die Bronchialwege verschmälern oder erweitern [17]. Im klinischen Alltag ist die konventionelle Röntgenuntersuchung zur primären Beurteilung der Lungen in Bezug auf Infiltrat, Dystelektase, Neoplasie oder röntgendichte Fremdkörper nicht mehr wegzudenken. Zur besseren Differenzierung und zur genaueren anatomischen Einordnung ist eine Schnittbildgebung, insbesondere eine CT, indiziert. Diese erlaubt auch eine dynamische Untersuchung der Atemwege, z. B. durch die Ergänzung einer Untersuchung in forcierter Exspiration.

Fremdkörper können die Bronchialwege teilweise oder vollständig verlegen. Ein röntgendichter Fremdkörper zeigt sich als deutlich transparenzverminderte Opazität. In der Konsequenz findet man distal des Fremdkörpers dystelektatische Lungenareale, die man an einer Volumenminderung und einer streifigen, retikulären/peribronchovaskulären Zeichnungsvermehrung erkennt. Eine vollständige Atelektase führt zu einer vollständigen, flächigen Verschattung eines Lungenareals sowie Retraktionen des ipsilateralen Zwerchfells und der perifokalen Pleura.

Infiltrat kann sich sowohl im interstitiellen Raum als auch in den Bronchien ausbreiten. Interstitielles Infiltrat zeigt sich konventionell radiologisch als retikuläre/peribronchovaskuläre Zeichnungsvermehrung ohne Volumenminderung des entsprechenden Lungenareals. Alveoläres Infiltrat erkennt man an der alveolären, fleckig erscheinenden Verteilung der Transparenzminderung und einem positiven Aerobronchogramm, einem Verschattungsmuster mit teils ausgesparten/belüfteten Bronchialwegen.

#### Endobronchialer Tumor

Es gibt verschiedene benigne und maligne endobronchiale Neoplasien ohne peribronchiale Tumorausdehnung. Das bronchiale Karzinoid findet sich häufig an einem größeren Bronchus wie einem Haupt- oder Lobärbronchus. Konventionell radiologisch ist es in frühen Stadien häufig nicht zu erkennen. Aber auch schnittbildgebend sind sie aufgrund der oblique zu den klassischen Rekonstruktionsebenen verlaufenden Bronchialwege ohne Bildnachverarbeitung in den frühen Stadien sehr schwer zu erkennen. In späteren Stadien zeigen sie sich als perihiläre, scharf begrenzte Opazität und durch sekundäre Zeichen wie eine Atelektase aufgrund einer Bronchialstenose. Daher werden diese Tumoren häufig erst diagnostiziert, wenn sie symptomatisch [17].

#### Zentraler Lungentumor

**Figure 6:**
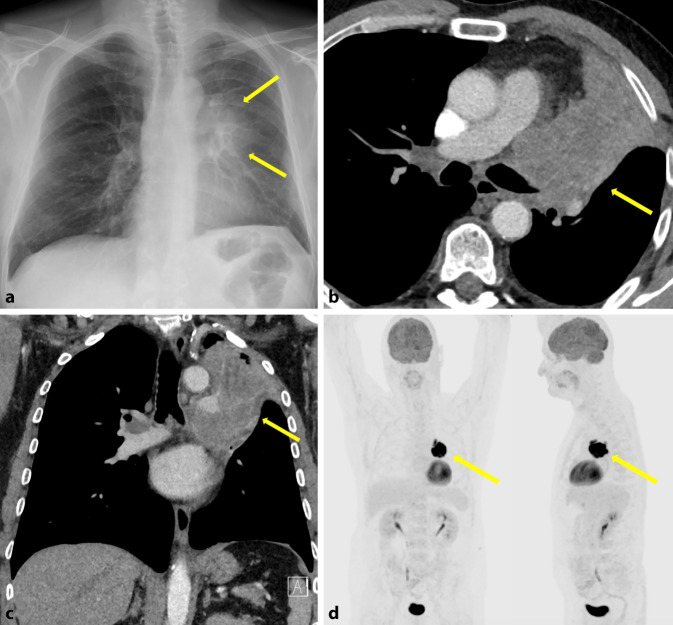

Lungenkarzinome werden histologisch in kleinzellige (SCLC) und nichtkleinzellige Lungenkarzinome (NSCLC) unterteilt. Die Hauptvertreter des NSCLC sind das Plattenepithelkarzinom, das Adenokarzinom und das großzellige Lungenkarzinom. Während es radiologisch nahezu unmöglich ist, die Entität des Lungenkarzinoms zu unterscheiden, so zeigt sich das SCLC häufig als (peri-)hiläre Opazität (Abb. 6). Da Lungenkarzinome meist erst im fortgeschrittenen Stadium symptomatisch werden, findet man konventionell radiologisch begleitend oft einen verbreiterten ipsi- oder auch kontralateralen LH, ein verbreitertes Mediastinum und einen Begleiterguss. Die Schnittbildgebung hingegen spielt im Staging der Lungentumoren und zur Evaluation der Operabilität und Operationsmethode eine wichtige Rolle. Auch wenn es mit den modernsten Spiral-CT und Bildnachverarbeitungen weiterhin schwierig bleibt, den einfachen Kontakt von einer Infiltration der perifokalen bronchialen und vaskulären Strukturen zu unterscheiden, zeigten Studien, dass die Positronen-Emissions-Tomographie kombiniert mit der CT (PET/CT) den T‑Status zu 82 % und den N‑Status zu 91 % richtig vorhersagen konnte [7, 17]. Obwohl hiläre Lymphknotenmetastasen die Operabilität nur selten beeinflussen, verschlechtert sich die Prognose deutlich. Im Stadium T1N0 ist häufig eine Segmentektomie, zum Erhalt von so viel Lungenparenchym wie möglich, der Therapieansatz [17].

Die CT wird weithin als primäre Standard-Bildgebungsmethode zur Beurteilung des Nodalstatus eingesetzt. Sie ist jedoch unzuverlässig, wenn es darum geht, den Lymphknotenbefall allein anhand von Größenkriterien zu beurteilen. Die Sensitivität und Spezifität der CT für die Stadieneinteilung von Lungenkrebs sind relativ gering, nämlich nur 57 % bzw. 82 % [27]. Das Größenkriterium der morphologischen MRT ist ebenso genau wie die CT.

**Figure 7:**
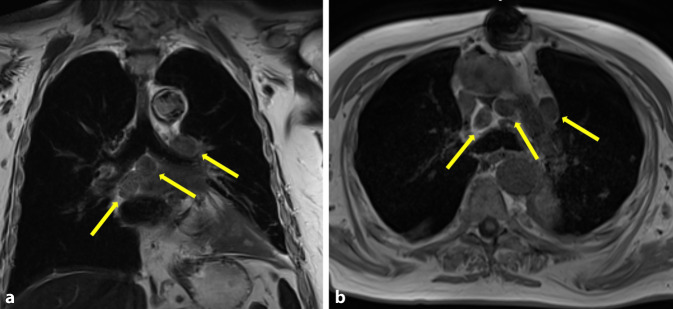

Verschiedene Studien konnten zeigen, dass die MRT bei der Charakterisierung der Lymphknoten der PET/CT fast ebenbürtig ist. So zeigten Ohno et al. [18], dass die STIR-Turbo-SE-Bildgebung zur quantitativen und qualitativen Beurteilung des N‑Stadiums bei Patienten mit nichtkleinzelligem Lungenkrebs mindestens so valide ist wie die PET/CT (Abb. 7). Darüber wiesen diverse Studien nach, dass die diffusionsgewichtete Bildgebung („diffusion-weighted imaging“, DWI) im Vergleich zur PET/CT eine hohe Spezifität für das N‑Staging von NSCLC aufweist und das Potenzial hat, eine zuverlässige alternative nichtinvasive Bildgebungsmethode für das präoperative Staging von mediastinalen und hilären Lymphknoten bei Patienten mit NSCLC zu sein [23, 29].

#### Seltene Neoplasien

Sehr selten können auch Sarkome am Lungenhilus entstehen. Sarkome sind maligne Tumoren, die aus mesenchymalen, Binde- und Stützgeweben entstehen. Sie können potenziell an jedem Ort des Körpers auftreten, wobei sie verdrängend wachsen und eher spät infiltrativ werden.

## Pitfalls

Radiologische Fehler fallen prinzipiell in zwei Kategorien: Detektierungsfehler und Interpretationsfehler. Das Röntgen-Thorax ist aufgrund von Überlagerungen, die unweigerlich bei der zweidimensionalen (2D) Darstellung dreidimensionaler (3D) Strukturen des Körpers entstehen, anfällig für beide Fehlerkategorien. Vor allem in bestimmten anatomischen Räumen, wie dem LH, ist das Fehlerpotenzial erhöht. Entweder werden Pathologien schlichtweg übersehen oder aber mit einer falschen Diagnose oder Normvariante verknüpft. So ist der häufigste Fehler bei der Interpretation des Röntgen-Thorax das übersehene Lungenkarzinom [6]. Aber auch bei der Interpretation der sensitiveren Schnittbildgebung ist die Kenntnis häufiger struktureller Fehlerquellen wichtig. Einige *Pitfalls* sollen im Folgenden besprochen werden.

### Konventionelle Röntgenaufnahme

Das Röntgen-Thorax wird bei Intensivpatienten täglich zur Statuserhebung eingesetzt. Es ist eine Herausforderung, diese Liegendaufnahme streng orthogonal und nicht rotiert anzufertigen. Durch einen nichtorthogonalen Strahlengang können verschiedene Pathologien vorgetäuscht werden. Beispielsweise können die LH bzw. die Mediastinalsilhouette vergrößert erscheinen. Da das Herz aufgrund des a.-p.-Strahlengangs größer wirkt, als es ist, kann so eine Kardiomegalie und eine pulmonale Hypertonie suggeriert werden. Außerdem kann ein PE größer oder kleiner und Lungenareale vermehrt oder vermindert belüftet erscheinen. Auch eine rotationsbedingte Verschiebung der azygoösophagealen Linie kann eine mediastinale Raumforderung vortäuschen [6].

Durch Hautfalten wird das Weichteil verdickt und es entsteht eine lineare Transparenzminderung durch Aufsummierung. Insbesondere in der Peripherie kann eine solche Hautfalte einen Pneumothorax suggerieren. Am LH bzw. paramediastinal können große vaskuläre Strukturen wie die Aorta oder das Mediastinum insgesamt verbreitert erscheinen. Auch bei der Bildverarbeitung können relevante Fehler entstehen. So können Patienten oder Seiten vertauscht werden. Durch eine versehentlich gespiegelt gespeicherte Aufnahme kann beispielsweise fälschlicherweise ein Situs inversus diagnostiziert werden. Im schlimmsten Fall kann es aber auch zu Eingriffen an der falschen Körperseite kommen.

### Schnittbildgebung

Die Entwicklung der Spiral-CT und die Möglichkeit der multiplanaren (MPR) und 3D-Rekonstruktion ist ein Meilenstein in der nichtinvasiven Diagnostik von Atemwegserkrankungen. Obwohl axiale Bilder die Sensitivität verglichen mit der konventionellen Röntgenuntersuchung bereits deutlich erhöhen, ist es wichtig, ihre Einschränkungen zu kennen. Es können subtile Atemwegsstenosen übersehen werden, aber auch die Lymphknotengröße oder die kraniokaudale Ausdehnung von Neoplasien aufgrund ihrer obliquen Lage zur axialen Schicht unterschätzt werden. Die MPR verbessert das Verständnis der teils komplexen anatomischen Strukturen und der Krankheitsausdehnung deutlich [2, 30].

## Fazit für die Praxis

- Obwohl der Lungenhilus (LH) kein Organ an sich darstellt, so hat er doch eine wesentliche Bedeutung, da er die Kommunikation zwischen Lunge und restlichem Körper ermöglicht.
- So finden sich am LH die zentralen Strukturen zur Versorgung der Lungen.
- Die moderne medizinische Bildgebung, insbesondere die Computertomographie (CT), erlaubt die frühzeitige Charakterisierung von Pathologien des LH.
- Dieses nichtinvasive Verfahren unterstützt eine schnelle und gezielte, interdisziplinäre Diagnostik, die vor allem in der Notaufnahme und in der stationären Patientenversorgung heutzutage nicht mehr wegzudenken ist.
- Genaue anatomische Kenntnisse sind jedoch für die korrekte radiologische Beurteilung unabdingbar.
